# Thalidomide‐induced teratogenesis: History and mechanisms

**DOI:** 10.1002/bdrc.21096

**Published:** 2015-06-04

**Authors:** Neil Vargesson

**Affiliations:** ^1^ School of Medical SciencesInstitute of Medical Sciences, University of AberdeenForesterhillAberdeenUnited Kingdom

**Keywords:** angiogenesis, reactive oxygen species, cell death, cereblon, actin cytoskeleton, limb development, *Fgf8*, *Shh*, phocomelia, vascular transition

## Abstract

Nearly 60 years ago thalidomide was prescribed to treat morning sickness in pregnant women. What followed was the biggest man‐made medical disaster ever, where over 10,000 children were born with a range of severe and debilitating malformations. Despite this, the drug is now used successfully to treat a range of adult conditions, including multiple myeloma and complications of leprosy. Tragically, a new generation of thalidomide damaged children has been identified in Brazil. Yet, how thalidomide caused its devastating effects in the forming embryo remains unclear. However, studies in the past few years have greatly enhanced our understanding of the molecular mechanisms the drug. This review will look at the history of the drug, and the range and type of damage the drug caused, and outline the mechanisms of action the drug uses including recent molecular advances and new findings. Some of the remaining challenges facing thalidomide biologists are also discussed. Birth Defects Research (Part C) 105:140–156, 2015. © 2015 The Authors Birth Defects Research Part C: Embryo Today: Reviews Published by Wiley Periodicals, Inc.

## Part one: History and Thalidomide Embryopathy

**Figure 1:**
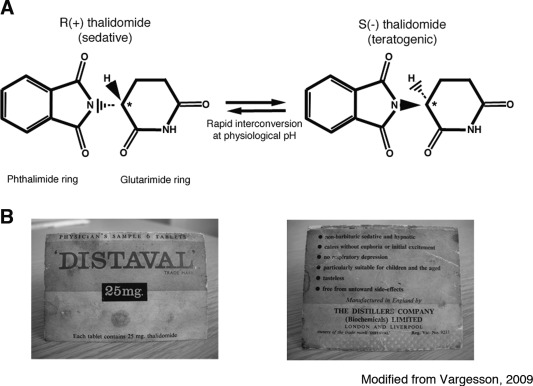
Structure of thalidomide enantiomers and packaging. A: Thalidomide is a stereo‐isomer and can exist in two enantiomeric states, depending on the state of the chiral carbon (see asterisk) allowing each form to have slightly different structural moieties. Both enantiomers, R and S, can rapidly interconvert (racemize) in body fluids and tissues and form equal concentrations of each form. B: Thalidomide was sold/distributed as a racemic mix of both enantiomers and called “Distaval” in the UK. These images are from an actual packet of “Distaval,” which was a Physician's Sample and given to women in early pregnancy. Note the safety advice on the packet. Reproduced from Vargesson, BioEssays, 2009,31,1327–1336.

Thalidomide was released in the late 1950's as a nonaddictive, nonbarbiturate sedative by the German pharmaceutical company, Chemie‐Grunenthal (Fig. 1). Thalidomide was very effective and quickly discovered to also be an effective anti‐emetic and used to treat morning sickness in pregnant women. Thalidomide was marketed and distributed in 46 countries around the world using different names. For example, the drug was known as Distaval in the UK and Australia, but was called Softenon in Europe and Contergan in Germany (Vargesson, 2009, 2013). Thalidomide became one of the world's largest selling drugs, and was marketed heavily and advertised as completely safe (Vargesson, 2009; Fig. 1B) right up until it was eventually banned in November, 1961. Indeed, sample packets of the drug were given out to physicians to distribute freely to patients suffering from morning sickness (Fig. 1B). Precisely how many women were given the drug will never be known.

Soon after thalidomide's release, reports surfaced of patients developing peripheral neuropathy after taking the drug. Reports of occurrences of severe birth defects affecting multiple body systems were also coming to light, that initially were not linked to, or were denied to be due to thalidomide. It was not until 1961 that thalidomide was confirmed by two independent clinicians, Lenz in Germany and McBride in Australia, to be the cause of the largest man‐made medical disaster in history (McBride, 1961; Lenz, 1962) with huge numbers (over 10,000) of severe birth defects in children. In addition, there were reports of increased miscarriage rates during this period (Lenz, 1988; Vargesson, 2009, 2013; McCredie, 2009; Stephens, 2009).

Thalidomide was subsequently withdrawn from the UK in Nov 1961 and by 1962 from most of the world. As a result, the incidence and occurrence of these severe birth defects was then not seen.

Whether this disaster could have been prevented remains unclear. The packaging and information of contraindications of drugs was minimal when compared to the way drugs are sold today (Fig. 1B). More importantly, the state of drug testing in the 1950s and 1960s was different when compared to today's standards. According to Grunenthal, the testing of thalidomide was carried out using the standards of the time (http://www.contergan.grunenthal.info/grt‐ctg/GRT‐CTG/Die_Fakten/FAQ/152700081.jsp). It remains a matter of contention/debate as to precisely what testing was carried out and, as importantly, when Grunenthal became aware of thalidomide being a human teratogen and whether the disaster could have been prevented sooner.

Thalidomide was not approved or licensed for use in the USA between 1957 and 1962, despite pressure to do so. An FDA physician, Frances Kelsey, responsible for approving drug licenses, had concerns about the safety of the drug, given the reports of peripheral neuropathy in patients taking the drug, but was also concerned about effects during pregnancy. Dr Kelsey was subsequently awarded the President's Award for Distinguished Federal Civilian Service by President John F. Kennedy for averting a thalidomide disaster in the USA (Vargesson, 2009, 2013).

The thalidomide disaster completely changed the way drugs are tested. In fact, articles written by Dr Frances Kelsey provided the foundation for modern day drug testing (Kelsey, 1988; Vargesson, 2013). Moreover, the thalidomide disaster demonstrated for the first time that species differences exist in drug reaction/response. Mice, traditionally used to screen for drug action, are less sensitive to thalidomide than other species like non‐human primates, rabbits, etc. (Merker et al., 1988; Stephens et al., 2000; Vargesson, 2013). Why mice are less sensitive to the drug remains unclear. Since the disaster, drug screening policies have changed to incorporate several species as well as *in vitro* tests, and there has not been a repeat of the disaster.

The evidence that thalidomide causes birth defects is now undoubted. Multiple animal species have been shown to be susceptible to thalidomide damage, including non‐human primates, rabbits, armadillos, Xenopus, marsupials, hamster, chicken, zebrafish, marine fish, hydra, and even bacteria (Fort et al., 2000; Vargesson, 2013; Hartmann et al., 2014). The thalidomide disaster of 1957‐61 highlighted, for the first time, that species differences do exist in the reaction to drugs.

Oddly, no individual nor Grunenthal itself was successfully prosecuted over the disaster. There are reports that Grunenthal may have entered into an agreement with the German Government and the German Federal Prosecutors offices to withdraw a criminal prosecution against them (http://www.theguardian.com/society/2014/nov/14/‐sp‐thalidomide‐pill‐how‐evaded‐justice). It took until 2012 before Grunenthal finally offered an “apology,” but stopped short of admitting liability, yet little compensation has yet been paid to thalidomide damaged survivors outside Germany (http://www.contergan.grunenthal.info/grt‐ctg/GRT‐CTG/Stellungnahme/Rede_anlaesslich_Einweihung_des_Contergan‐Denkmals/224600963.jsp). Grunenthal no longer produces thalidomide, but today is one of the world's largest Pharmaceutical companies producing drugs for pain relief.

The survivors of the thalidomide disaster have severe handicaps, and many of the survivors are experiencing early onset age related issues, such as osteoarthritis, joint mobility issues and coronary heart disease, making life more difficult (Miller and Stromland, 1999; Newman, 1999; Smithells and Newman, 1999; McCredie, 2009; Vargesson, 2009, 2013). In some countries the distributors of the drug, for example, Distillers in the UK and Australia (now Diageo), have been sued for compensation by the survivors. Compensation has been received from such distributors. In addition Government support has been given to survivors. However the amount of support varies between countries.

Today, thalidomide is used to successfully treat a wide range of medical conditions, which include leprosy, multiple myeloma, and cancers, as well as Crohn's disease, HIV, and others. Thalidomide use is carefully monitored using successful schemes like the S.T.E.P.S. (System for Thalidomide Education and Prescribing Safety_program (Zeldis et al., 1999; Uhl et al., 2006), which monitors patients to ensure they are not pregnant while receiving treatment.

However, tragically a new generation of thalidomide survivors has occurred in Brazil (Castilla et al., 1996; Schuler‐Faccini et al., 2007; Vianna et al., 2013). Thalidomide is used to treat complications of leprosy in Brazil, which is sadly common and debilitating. Unfortunately the drug is given to patients who share the medicine with others, and who do not understand or are not informed of the dangers, and damaged children are born. The damage to these children is similar to the damage seen in children between 1957 and 1962.

## Thalidomide Embryopathy

Between 1957 and 1962, thalidomide caused severe birth defects in over 10,000 children. Almost any tissue/organ could be affected by thalidomide. Indeed a detailed UK Government sponsored report in 1964 (UK Government Report, 1964) detailed that almost all the tissues and organs of the body could be affected by the drug. Some hallmark/consistent features were identified by German, UK, and also Australian physicians (Lenz and Knapp, 1962; Smithells, 1962; Spiers, 1962; Taussig, 1962; Nowack, 1965; Smithells, 1973; McBride, 1976; Ruffing, 1977; Newman, 1985; Newman, 1986; Smithells and Newman, 1992; McCredie, 2009). Damage was primarily seen to the limbs (upper limbs more commonly affected than lower limbs), face, eyes, ears, genitalia, and internal organs, including heart, kidney, and gastrointestinal tract. The vertebral column was also affected in some survivors and occurrence of facial palsies was also documented.

Due to the wide range of damage and conditions that thalidomide exposure can cause, the damage is usually referred to collectively as thalidomide embryopathy or thalidomide syndrome, as some of the damage is not unique to thalidomide embryopathy and can be seen in other human conditions, for example Duane syndrome (Smithells and Newman 1992; Miller and Stromland, 1999). Thalidomide embryopathy is a severe condition and affects many tissues, all of which can occur independently in humans but rarely together.

Infant mortality in babies born with severe thalidomide embryopathy is as high as 40%, quite likely due to internal organ damage (Smithells and Newman, 1992; Vargesson, 2013). Furthermore, many babies with these malformations are likely to have died *in utero* and been miscarried or stillborn. The true numbers of babies affected by thalidomide will likely never be known.

The hallmarks/consistent features of thalidomide embryopathy were identified by examining the most severely affected children to determine a diagnostic criteria, which is still used today (Smithells and Newman, 1992). Recently the diagnostic criteria was re‐examined following a World Health Organization sponsored Advisory Panel and a new, broader diagnostic system has been suggested (www.who‐umc.org/graphics/28280.pdf *)* .

### Limb defects

Damage to the limbs are one of the most common and studied features of thalidomide embryopathy.

#### Upper limb

Phocomelia remains the most striking limb deformity caused by thalidomide, and remains the stereotypical image of thalidomide embryopathy. Phocomelia occurs through a severe shortening of the limb/s, due to proximal elements (long bones) being reduced or missing and leaving distal elements (handplate) in place. Phocomelia ranges in severity, from severe, where long bones are missing with just a flipper‐like structure consisting of digits/handplate articulating with the body, to less severe forms exhibiting a shortening of the long bones and normal distal bones. Radial dysplasia (loss of the radius and thumb) is also seen in thalidomide survivors (Lenz and Knapp, 1962; Spiers, 1962; Taussig, 1962; Ruffing, 1977; Smithells, 1973; Newman, 1985; Smithells and Newman, 1992). The range and type of upper limb deformities induced by thalidomide exposure exhibit a characteristic pattern. The thumb is the first structure to be affected, followed by the radius, humerus, and lastly the ulna (Lenz and Knapp, 1962; Nowack, 1965; Smithells and Newman, 1992; McCredie, 2009; Miller et al., 2009). The majority of thalidomide survivors exhibit some form of limb deformity, which are usually reduction defects and most often symmetrical. Indeed symmetrical limb defects remain one of the hallmarks for the diagnosis of thalidomide embryopathy. However, several studies indicate that unilateral limb defects can occur in thalidomide survivors and differences in severity can also occur between each side of the body—so does not have to be symmetrical (Lenz and Knapp, 1962; UK Government Report, 1964; Schmidt and Salzano, 1980; Schmidt and Salzano, 1983).

#### Lower limb

The lower limbs can also exhibit thalidomide‐induced damage. Phocomelia or Amelia are seen, as well as reduction in the long bone lengths. However, abnormalities of the lower limbs are seen less commonly than those of the upper limbs, with lower limb deformities on their own occurring, but being very rare. The femur is the bone most often affected in the lower limb, and similarly to the ulna, the fibula is usually the final bone to remain normal (Nowack, 1965; Hamanishi, 1980; Lenz, 1988; Smithells and Newman, 1992; Miller and Stromland, 1999; McCredie, 2009).

Polydactyly (extra digits) was also observed in hands and/or feet of thalidomide survivors in reduced limbs, as well as in combination with phocomelia (UK Government Report, 1964; McBride, 1961; Smithells and Newman, 1992; Vianna et al., 2013). The ability of thalidomide to cause severe limb reduction or loss of proximal elements, but to then duplicate distal digits, adds to the mystery of thalidomide‐induced embryopathy.

Why the upper limbs are more affected in thalidomide survivors is unclear. We do know the lower limbs form slightly after upper limbs, and we know that thalidomide has a short half‐life of activity, thus it is possible a single dose in the early stages of pregnancy could affect the upper limbs, whereas multiple doses over a few days may be required to affect the later forming lower limbs.

The severity of limb damage seen in thalidomide survivors can vary between each side of individuals. Some cases exhibit bilateral disorders and others have a more severely affected limb on one side of the body. More strikingly, there are several reports of unilateral limb damage in thalidomide survivors, though these cases are rare (Lenz and Knapp, 1962; Somers, 1962; UK Government Report, 1964; Schmidt and Salzano, 1980; Schmidt and Salzano, 1983).

### Shoulder and hip joints

Characteristic shoulder and hip joint damage occurs in thalidomide embryopathy. For example, the acromioclavicular joint of the shoulder is more prominent, and sharpened in appearance, when the shoulder is damaged through thalidomide exposure (Newman, 1977). The hip joint can be hypoplastic or, in some cases, completely absent, as is also true for the pubic bone (Newman, 1977).

### Eye and ear damage

Eye and ear damage (internal and external) is another hallmark used to diagnose thalidomide embryopathy. Eyes and ears develop in the embryo from week 4/5 until around week 8/9, which is around the same time the limbs are rapidly growing. Thalidomide can cause small eyes (micropthalmia), anophthalmos (absence of the eyeball), and poor vision. Eye defects also include aberrant or no lacrimation (tear formation), coloboma, and strabismus (Smithells, 1962; Cullen, 1964; James, 1965; Newman, 1977; Schmidt and Salzano, 1980; Kelsey, 1988; Miller and Stromland, 2011). Ocular defects can occur unilaterally, although there may still be poor vision in the unaffected eye (Smithells, 1962). Abnormalities in eye movement are also observed and usually occur in conjunction with ear defects and facial muscle weakness (Smithells and Newman, 1992).

Ear defects in thalidomide survivors are usually symmetrical, ranging from absence of the outer (anotia) to reduction of the outer ear (microtia; Ruffing, 1977; Smithells and Newman, 1992). Deafness or reduced hearing, as well as cranial nerve palsies in addition to ear defects, are also observed in some thalidomide survivors (Smithells and Leck, 1963; Livingstone, 1965; Newman, 1977; Ruffing, 1977; Newman, 1985; Miller and Stromland, 1999).

### Facial damage

Another key hallmark of thalidomide embryopathy is the presence of an enlarged nevus or hemangioma at birth (also known as a “storkmark”; Lenz and Knapp, 1962; Lenz, 1988; Smithells and Newman, 1992; McCredie, 2009; Stephens, 2009). The hemangioma is usually observed in the centre of the forehead and can extend over the nose to the upper lip. This is a capillary hemangioma and is temporary, and usually disappears within 2‐3 years of birth (Newman, 1985, 1986; Smithells and Newman, 1992). Facial palsy and facial asymmetry are also associated with thalidomide embryopathy, likely due to weakened facial muscles and facial nerve damage (Newman, 1985, 1986; Smithells and Newman, 1992; Miller and Stromland, 1999). Other facial problems linked to thalidomide damage include irregular teeth numbers/spacing, small jaws, cleft palate and cleft lip, and small noses (Newman, 1985, 1986; Smithells and Newman, 1992).

### Vertebral column

Various vertebral column issues have been reported in thalidomide survivors, including irregular vertebral spacing; fusion of vertebrae particularly in the lower spinal column, and in some cases progressive kyphosis which often requires surgical intervention throughout life (Ruffing, 1977; Smithells and Newman, 1992). Thalidomide survivors can have shorter stature than average, with the shortness due to shorter leg bones, independent of vertebral defects (Brook et al., 1977; Ruffing, 1977; Newman, 1985; Smithells and Newman, 1992).

### Internal organ damage

All of the internal organs can be affected following thalidomide exposure *in utero*. Damage seen includes malformations of the heart, kidneys, genitals, and gastrointestinal tract (Smithells and Newman, 1992; Ruffing, 1977; Lenz and Knapp, 1962). However, the precise incidence of such deformities is unknown, as such defects are not always outwardly seen or apparent, particularly if they do not present until later in life.

Defects of the heart are likely the cause for many of the intrauterine and post‐natal deaths. Urinary tract and kidneys also exhibit a range of life‐threatening conditions, including horseshoe, hypoplastic, rotated, and ectopic malformations (Nowack 1965; Newman, 1985, 1986; Smithells and Newman, 1992). Genital defects, both internal and external, have been reported in thalidomide survivors. These include absence of the testes, testicular abnormalities, and hypospadiasis in males, and in females malformations of the uterus and reproductive tract defects (Cuthbert and Speirs, 1963; Newman, 1985; Newman, 1986; Smithells and Newman, 1992). Other common problems associated with thalidomide exposure include anorectal stenosis, intestinal atresia, pyloric stenosis, and inguinal hernia (Lenz and Knapp, 1962; Somers, 1962; Cuthbert and Speirs, 1963; Nowack, 1965; Newman, 1985, 1986; Smithells and Newman, 1992)

### Nerve and CNS damage

There is evidence that some thalidomide‐damaged children have facial palsies, cranial nerve conduction problems, and an increased incidence of autism and epilepsy, which is only diagnosed later life (Soules, 1966; Smithells and Newman, 1992; Miller and Stromland, 1999; Miller et al., 2005). Thalidomide could affect developing neural pathways (Miller and Stromland, 1999), possibly by preventing angiogenesis in the brain (Hallene et al., 2006), resulting in cell death and loss of tissue. Animal model evidence indicates such damage can occur quite late in fetal development (Hallene et al., 2006).

## When Does Thalidomide Cause Damage to the Embryo?

**Figure 2:**
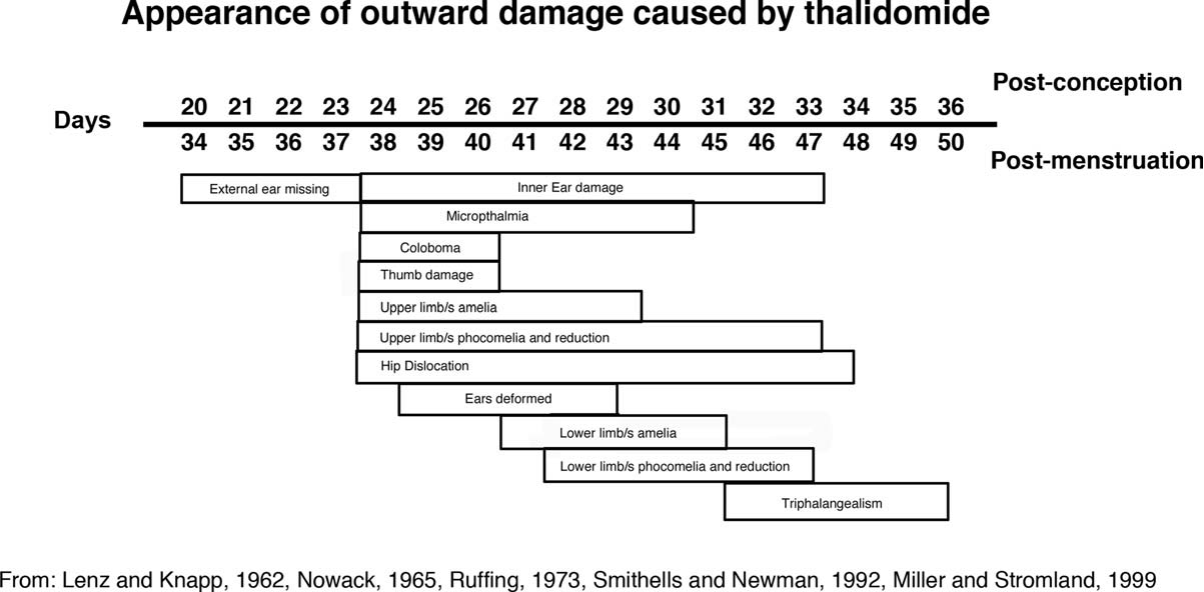
Time sensitive window of thalidomide embryopathy or the “critical period.” Chart indicating when the major outward appearing damage occurred in the embryo following thalidomide exposure.

Thalidomide causes damage to the forming embryo in a short time sensitive window also known as the “critical period.” The time sensitive window extends between days 20 and days 36 after fertilization (34–50 days after last menstrual cycle; Fig. 2; Lenz and Knapp, 1962; Nowack, 1965; Smithells and Newman, 1992; Miller and Stromland, 1999). Exposure before and after this time sensitive window is thought to not cause damage to the embryo (Newman, 1985, 1986; Lenz and Knapp, 1962; Smithells and Newman, 1992). However, early exposure to thalidomide in humans and rats induces miscarriage, and late fetal exposure in rats induces brain damage (James, 1965; Kajii et al., 1973). This indicates there is likely no safe time period to take the drug.

Reports indicate that a single 50 mg tablet of thalidomide during the time‐sensitive window is sufficient to cause birth defects in up to 50% of pregnancies. Some reports go even further and suggest it would be surprising if any embryo exposed to thalidomide during the time‐sensitive window was unharmed (Newman, 1985; Stromland and Miller, 1992; Smithells and Newman, 1992). Indeed a detailed 1964 UK Government report into the thalidomide disaster in the UK indicates that a few children were born unharmed or without outward damage to mothers with confirmed exposure to the drug during pregnancy (UK Government Report, 1964). Whether these individuals had other problems, not obvious at birth, that became apparent later in life is unknown.

The time‐sensitive window of thalidomide teratogenicity was determined following interviews with parents of thalidomide affected children and their doctors (Lenz and Knapp, 1962; Nowack, 1965; Ruffing, 1977; Lenz, 1988; Miller and Stromland, 1999; McCredie, 2009). From these interviews, dates of intake and exposure were ascertained and correlated to the damage seen in the infants, to determine the timing of damage. This window correlates with outward damage primarily and severe internal organ damage. Obviously any other damage that may not present until later in life could not be recorded. Exposure to thalidomide earlier in the time sensitive window results in more severe damage, as evidenced by outward signs, such as effects on the limbs, face, and genitals, and obvious internal damage, such as kidney failure (Fig. 2). Exposure towards the end of the window results in injuries to digits and/or eyes/ears.

The time‐sensitive window is concomitant with the period of rapid embryonic development (from 4 weeks onwards) with a lot of cell movement, proliferation, organogenesis blood vessel formation to allow all the organ and tissue development and also many different signaling pathways being active. Embryogenesis lasts until around week 10–11 when the embryo is fully patterned and formed. The remaining period of pregnancy allows the fetus, as it is now termed, to grow and mature. As thalidomide was used to treat morning sickness, which can occur from week 4 and last until week 12 in some women, and in others throughout pregnancy, this exposure coincides with the major developmental events underpinning the thalidomide disaster.

Given that the majority of studies in humans in the 1960's assessed damage in severely affected infants, and there are very few studies assessing the damage or late onset damage in adults, it is possible that the drug did cause far more damage than initially described.

Animal studies, particularly in nonhuman primates, show that following thalidomide exposure a range of damage is induced, which can vary between each of the litter‐mates (Merker et al., 1988; Ema et al., 2010; Vargesson, 2013). For example, one littermate could exhibit varying damage to all 4 limbs, another to one limb, and another just a tail anomaly (Merker et al., 1988; Ema et al., 2010; Vargesson, 2013). The mechanism underlying this variance in damage is unknown, but also hints that thalidomide damage in humans could be broader.

## Thalidomide‐Induced Damage Can Phenocopy some other Human Conditions

The damage thalidomide causes can vary widely between individuals (Lenz and Knapp, 1962; UK Government Report, 1964; Ruffing, 1977; Schmidt and Salzano, 1983; Smithells and Newman, 1992). How and why the drug caused such a range and variability in damage remains unclear, but likely includes individual differences in metabolism and clearance of the drug, as well genetic and environmental factors.

Some of the thalidomide induced damage is not unique to thalidomide embryopathy, but can be seen independently in other human conditions, such as facial palsies, Duane syndrome, and autism and limb reduction, for example, radial dysplasia (Smithells and Newman, 1992; Miller and Stromland, 1999; Lenz and Knapp, 1962). Indeed, some of the damage the drug causes can often be confused for other human congenital malformations. This can make diagnosis challenging on occasion, particularly if the mother cannot recall or admit to using thalidomide during pregnancy. For example, both Okihiro Syndrome, characterized by limb reduction anomalies and Holt‐Oram Syndrome, typically presenting with heart and limb reduction deficiencies, can vary in severity and have been confused with thalidomide embryopathy (Kohlhase et al., 2003; Kohlhase and Holmes, 2004; Vargesson, 2013). As has another much rarer condition, Roberts Syndrome, also known as pseudothalidomide syndrome, given its striking phocomelia to all four limbs, facial damage, and internal organ damage. Roberts Syndrome is caused by a mutation in ESCO2, a gene involved in chromosome separation, cell division, and DNA repair (Bates, 2001; Schule et al., 2005; Vega et al., 2005; Whelan et al., 2012). A zebrafish model of Roberts syndrome indicates that ESCO2 loss prevents normal development by disrupting the cell cycle (Monnich et al., 2011). Finally, tetra‐amelia occurs in thalidomide survivors but can also occur in human populations via a homozygous mutation in the *Wnt3* gene (Niemann et al., 2004). Patients with *Wnt3* mutations also have facial and urogenital damage, also seen in some thalidomide survivors. Whether any of the genes associated with these human genetic conditions are targets of thalidomide to cause thalidomide‐induced embryopathy remains unclear. The similarity of thalidomide embryopathy to other human conditions highlights the difficulties faced in diagnosing conditions. With the advent of genetic testing, many conditions can now be tested for and ruled out.

Another important aspect that helps identify thalidomide embryopathy from genetic conditions is that thalidomide is not a mutagen and defects are not hereditary, that is, passed on to the next generation (Ashby et al., 1997; Ashby and Tinwell, 2002). Indeed a recent study in Sweden followed offspring from thalidomide survivors and found no evidence of any injuries or damage in their offspring (Stromland et al., 2002).

## Today

The most severely affected children exhibited limb malformations, facial, genital, and internal organ problems altogether, and the study of the most severely affected thalidomide babies/children, usually those with bilateral limb damage (Lenz and Knapp, 1962; Smithells and Newman, 1992), provided the basis for the diagnostic criteria used to diagnose thalidomide embryopathy today. However, patients with less severe damage or, for example, not exhibiting bilateral limb damage, were excluded. The precise range and severity of damage this drug caused and causes may never be known. However, with the ongoing thalidomide tragedy in Brazil, the thalidomide survivors, many of whom are children or young adults, offer the possibility of studying the damage caused and its progression, as well as late onset problems. Furthermore, in early 2014 the World Health Organization hosted a meeting of experts in thalidomide to re‐look at both the diagnostic criteria and produce a broader range of criteria in light of the Brazil tragedy, and also the increased use of thalidomide around the world, and to provide guidance on the drug's use and contraindications. The new diagnostic algorithm proposed (www.who‐umc.org/graphics/28280.pdf *)* will hopefully identify more victims of the drug and help them get better treatment/therapies.

## Part Two: Biochemistry And Mechanisms of Action

Thalidomide was marketed as a nonaddictive, nonbarbiturate sedative that would also be nonlethal if overdoses occurred (unlike barbiturates; Fig. 1B). The drug is a synthetic derivative of glutamic acid, a naturally occurring amino acid involved in important physiological processes, for example, brain neurotransmission and metabolism.

Thalidomide consists of two linked rings, a glutarimide and pthalimide ring (Fig. 1A). Thalidomide has a chiral carbon, which is unstable and allows two enantiomers to coexist, which can interswitch between the two states rapidly in bodily fluids and in water (Smith et al., 1965; Franks et al., 2004). One of the enantiomers is teratogenic, the S‐enantiomer. However as the drug can convert (racemise) between enantiomeric states, it is very difficult to make a stable form that is nonteratogenic. Thalidomide was sold as a racemic mix of both enantiomers (Fig. 1A) (Franks et al., 2004).

The parent molecule is believed to have an active half‐life of around 8–12 hr. The drug can be hydrolyzed in bodily fluids and can also be metabolized in the liver by cytochrome p450 enzymes (Fig. 1). The parent molecule is believed to be the cause of the teratogenesis; however, many breakdown products are found quickly after administration (at least 18; Smith et al., 1965; Franks et al., 2004) and some have been found to be teratogenic in *in‐vitro* embryo cultures suggesting a combination of parent molecule and breakdown products cause teratogenesis. (Lee et al., 2011).

Since thalidomide was banned and withdrawn in 1961/2, the drug has been discovered to have antiangiogenic, anti‐inflammatory, and anti‐myeloma roles. In fact, thalidomide was found to be useful in treating leprosy as early as 1965 and is now used to treat complications of the condition around the world (Sheskin, 1965). In 1994, thalidomide was demonstrated to possess anti‐angiogenic actions, which were suggested could be the cause of thalidomide embryopathy (D'Amato et al., 1994). Thalidomide is now used to treat some cancers. Thalidomide has also been demonstrated to be a potent inhibitor of the inflammatory response, through inhibiting the production of TNF‐α (Moreira et al., 1993). Indeed this action has made the drug very successful for treatments for conditions including Leprosy and Multiple Myeloma, Crohns disease, Behcets disease, HIV, lupus, and leprosy.

## Mechanisms of Action underlying Thalidomide Embryopathy

How does thalidomide cause the severe and wide range of damage in the embryo? The short answer is that it is still not fully understood. Over 30 separate models/theories for thalidomide embryopathy have been proposed over the past 50 years and are reviewed in detail elsewhere, that include DNA mutagenesis, effects on chrondrogenesis, nerve/neural crest toxicity, and inhibition of cell adhesion molecules (Stephens, 1988; Stephens and Fillmore, 2000; Stephens et al., 2000; Vargesson, 2009, 2013). Some studies present *in vivo* evidence and some are hypotheses, and any valid theory needs to be able to address the time‐sensitive nature of the drug action and the range and variability of the damage caused.

While these theories are interesting and have some merits the theories presently widely supported are thalidomide's antiangiogenic actions—the drug's ability to induce cell death and generate reactive oxygen species (ROS); the thalidomide binding target, *Cereblon*, a ubiquitin ligase, which if prevented from binding can reduce thalidomide induced damage in embryos (Stephens, 2009; Vargesson, 2009, 2013; Ito et al., 2011).

However, these theories are not necessarily mutually exclusive. Indeed, it is more than likely that all of the proposed theories are involved in some aspect of the cascade of events that thalidomide induces to cause birth defect. Moreover, other molecular targets have also recently been linked to thalidomide embryopathy.

## Thalidomide is antiangiogenic

Thalidomide was demonstrated in a landmark study to inhibit angiogenic vascularization of rodent corneas induced by fibroblast growth factor (GF) protein (D'Amato et al., 1994). This discovery led to the suggestion that thalidomide might cause its teratogenic damage by targeting embryonic blood vessels.

### Angiogenesis Is Essential for Embryogenesis

Blood vessels are essential for normal embryonic development. Blood vessels supply oxygen and nutrients to growing tissues and remove unwanted waste products. During embryogenesis, vessels form first by vasculogenesis and are then modified into the complex vascular tree required for embryonic and fetal growth and throughout adult life by angiogenesis (Vargesson, 2003). Angiogenesis is where the primitive vessels formed by vasculogenesis are elaborated upon where endothelial cells in the existing vessels proliferate and migrate to avascular areas in response to signals, including hypoxia or vascular endothelial growth factor. Once a new vessel tube has been made, the vascular tube recruits vascular smooth muscle cells, which stabilize the vessel. The smooth muscle coating is lost to allow endothelial cells to proliferate and migrate into new regions if signaled to do so (Vargesson, 2003). Blood vessels are rapidly changing throughout embryogenesis and organogenesis to accommodate the changes and growth of the embryo.

Blood vessels are essential for normal embryonic development, and vessel loss or disruption can unsurprisingly result in death or birth defect (Vargesson, 2003, 2009).

### Angiogenesis Is Targeted by Thalidomide in Embryonic Development and in the Adult

Thalidomide has multiple actions in the adult body and causes a variety and range of damage in the embryo. To determine how and which of its activities actually cause teratogenesis, stable, structural analogs of thalidomide were screened to determine their function, confirm which aspect of thalidomide action, antiangiogenesis or antiinflammation, results in teratogenesis, and study the resulting damage to gain insights into how the drug causes birth defect (Ng et al., 2003; Franks et al., 2004; Therapontos et al., 2009; Vargesson, 2009; Mahony et al., 2013).

Thalidomide can break down into many byproducts either hydrolytically or via liver metabolism (Franks et al., 2004). A range of byproducts of thalidomide, including CPS49, a tetrafluorinated analog based on 5′‐OH‐thalidomide, which is one of the breakdown products of thalidomide and thought to have antiangiogenic activities, were produced (Price et al., 2002; Ng et al., 2003; Franks et al., 2004; Vargesson, 2013). Fluorination confers stability and increased biological activity onto compounds (Ng et al., 2003; Franks et al., 2004).

**Figure 3:**
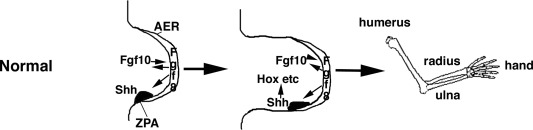
Embryonic limb development. Limbs form from a bud from the flanks of the embryo. In humans the upper limbs form around a day earlier (day 26) than the lower limbs (day 27). The limb bud consists of two key signaling centers. The apical ectodermal ridge (AER), a thickened epithelium lining the distal tip of the bud and separating the dorsal from ventral surface; and the zone of polarizing activity (ZPA) in the posterior‐distal mesenhcyme. The AER expresses Fgf8 which signals to the mesenchyme to induce Fgf10 and to the ZPA to induce and maintain Shh, which itself feeds back to maintain *Fgf8*. This feedback loop maintains cell proliferation and limb outgrowth and induces other genes, for example the *Hox* genes, which establish the pattern of the limb elements, humerus, radius, ulna, and handplate, as well as the soft tissues. The limbs grow out from specific regions of the flank of the embryo and as the limb grows out the limb is patterned proximally to distal, that is, humerus/femur are laid down before the radius, ulna/fibular, tibia, and then the handplate/footplate.

CPS49 was applied over the upper half of the chicken embryo as limbs are just forming (E2.5; Therapontos et al., 2009). Blood vessels were rapidly destroyed within 1 hr of exposure in the embryo and several hours before any changes in limb signaling gene expression and cell death was observed (Therapontos et al., 2009; Vargesson, 2009, 2013). Reduction in limb area was seen after 6 hr. After 24 hr, truncated or severely shortened limbs were observed and in addition, increased cell death was seen, and loss of *FGF* (in both the apical ridge and mesenchyme) and Sonic hedgehog (*Shh*) signaling was apparent (Fig. 2). *FGF* and *Shh* are key signaling molecules in the developing limb, controlling patterning and outgrowth (Fig. 3).

**Figure 4:**
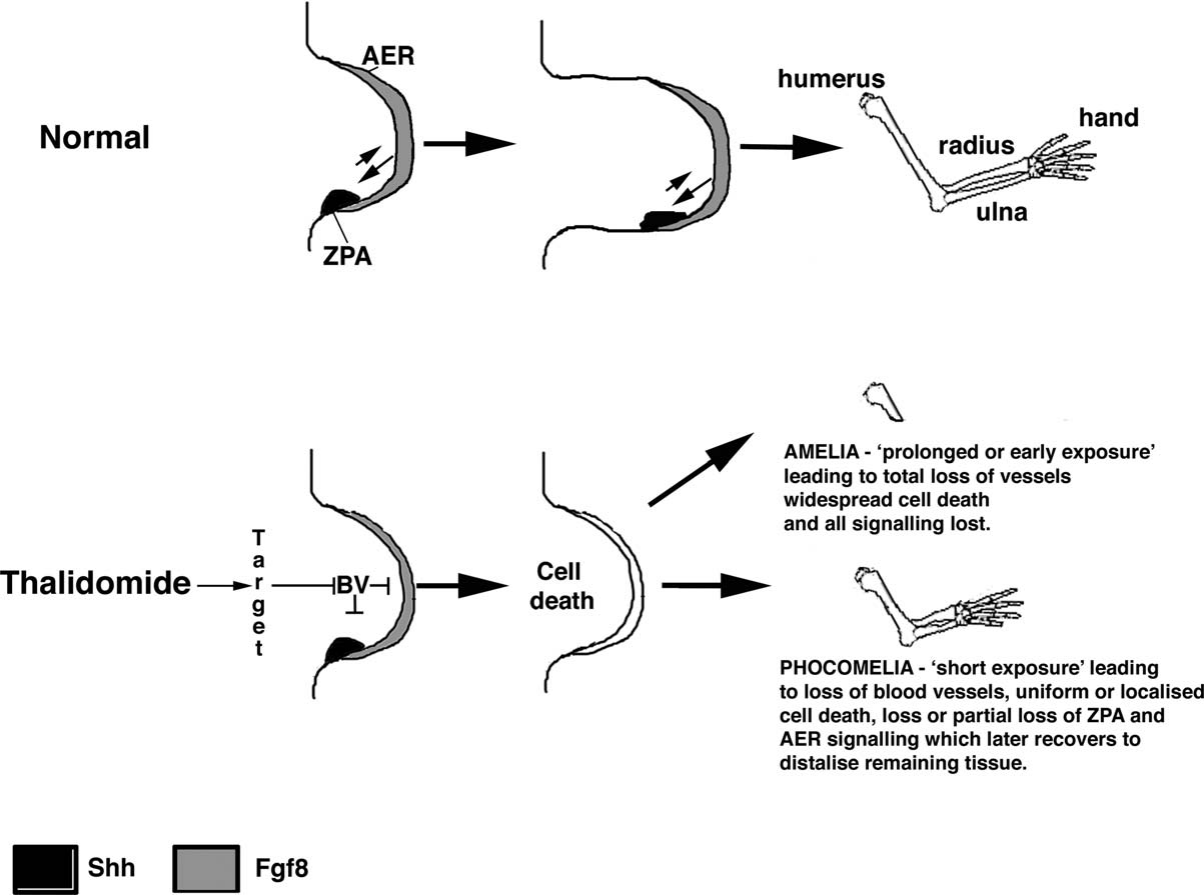
How does thalidomide induce Phocomelia? Phocomelia is the striking characteristic of thalidomide embryopathy which remains the public image of the condition and disaster. Phocomelia, the loss of or severe shortening of the limbs long bones, is rarely seen in other conditions. How can thalidomide cause Phocomelia? Thalidomide and some of its analogs have been shown to reduce or inhibit *FGF* (and *Shh*) expression in developing limbs of rabbits and chickens (Hansen et al., 2002; Therapontos et al., 2009; Knobloch et al., 2011). The expression of these genes returns after a few days (Hansen et al., 2002; Knobloch et al., 2011). Moreover, reduction of FGF signaling in developing mouse limbs can cause phocomelic‐like limb injuries through causing increased cell death in proximal tissues (Mariani et al., 2008). Thalidomide exposure induces cell death in chicken embryo limbs (Knobloch et al., 2007), as does CPS49 an antiangiogenic analog of thalidomide (Therapontos et al., 2009). It has been proposed that thalidomide destroys blood vessels, resulting in cell death and loss of the limb signaling pathways (Therapontos et al., 2009). The proximal tissue will be lost permanently and unable to be replaced, due to a combination of the limb being unable to replace the huge number of cells lost, but also the molecular environment in the limb that patterned the proximal elements will no longer be present or be able to be recapitulated (Mahony and Vargesson, 2013). Recovery or reactivation of *FGF* signaling could allow the limb to recover, but only distal structures would form from the remaining tissue, as the tissue is so close to the apical ridge and influenced by distal signals only. Perhaps a misregulation of these signals could also explain the polydactyly seen in some thalidomide survivors? If the exposure to thalidomide is prolonged or occurred just as the limbs were forming, this could lead to total vessel loss, widespread cell death, and a complete loss of *FGF* (and *Shh*) signaling, which is unable to recover and result in Amelia (no limb).

Thalidomide has also been reported to cause loss of *FGF* and *Shh* signaling in chick and rabbit embryo limbs and in zebrafish embryos (Hansen et al., 2002; Ito et al., 2010; Knobloch et al., 2011). In CPS49 treated chicken embryos, phocomelia‐like limbs were observed 7 days after just a single exposure (Fig. 4). Just like Thalidomide, CPS49 acts in a time‐sensitive window. Early exposure resulted in severely truncated limbs, even Amelia, whereas later exposure caused less severe damage, for example loss of a digit or digit tips. In fact the damage caused by CPS49 in chicken embryos was consistent with damage seen in other studies using thalidomide (Somers, 1962; Boylen et al., 1963; Merker et al., 1988; Tamilarasan et al., 2006; Therapontos et al., 2009; Vargesson, 2009, 2013; Ema et al., 2010).

Similarly, in zebrafish embryos, blood vessels in the developing spinal cord are stunted and lost rapidly following CPS49 treatment. CPS49 was also shown to prevent Human Umbilical Venous Endothelial Cells (HUVEC) in culture from proliferating and migrating to form new tubes, through preventing actin and tubulin cytoskeletal changes and inhibiting filopodial tip cell formation and migration (Therapontos et al., 2009).

Studies using CPS49, an antiangiogenic analog of thalidomide, demonstrated that thalidomide's antiangiogenic action can cause its teratogenic actions in a time sensitive manner (Therapontos et al., 2009).

### Why Are Some Vessels Targeted While Others Are Apparently Unharmed?

Blood vessels undergoing angiogenesis lose their vascular smooth muscle coats to allow endothelial cells to proliferate and migrate to form new tubes (Vargesson, 2003, 2009, 2013). Vessels with smooth muscle coats are quiescent and not undergoing angiogenesis. Using *in‐vitro* rat and mouse aortic ring culture assays, CPS49 was demonstrated to destroy vessels without smooth muscle, but blood vessels possessing smooth muscle coats were protected (Therapontos et al., 2009). This indicates that newly formed and forming blood vessels without smooth muscle coats could be susceptible to thalidomide. In the chicken embryo at the time the drugs are applied to the embryo, the developing limb vasculature does not possess smooth muscle (Vargesson and Laufer, 2001; Therapontos et al., 2009). In fact, the developing limb does not exhibit smooth muscle coverage of vessels until quite late in limb development (Vargesson and Laufer, 2001; Vargesson, 2009, 2013). In contrast to the limb, at the time of exposure (E2.5), the majority of the vessels in the embryo, including the microvessels in the brain and vessels in the neck and body, possess smooth muscle coats, intimating that these vessels are quiescent and protected at this time (Therapontos et al., 2009; Vargesson, 2009). This observation correlates well with the range of limb defects seen, depending on the timing of exposure. Severe defects are seen after early exposure, but by the time smooth muscle has appeared on the limb vasculature, defects are restricted to digits (Therapontos et al., 2009). Furthermore, when the drug was applied to very young embryos (E1.5), when most tissues are being vascularized and organogenesis is occurring, embryos died (Therapontos et al., 2009).

Several nonantiangiogenic analogs and hydrolysis products of thalidomide tested using the same assays were found to not be teratogenic or obviously harmful to chicken embryos (Therapontos et al., 2009). This indicates that the antiangiogenic action of the drug can cause a wide range of limb defects in a time‐dependent manner.

CPS49 in chicken embryos causes loss of vessels before changes in limb signaling pathways. In zebrafish embryos, CPS49 causes rapid loss of vessels, which from HUVEC studies is due to inhibition of actin cytoskeletal dynamics (Therapontos et al., 2009). Other studies in zebrafish have demonstrated that thalidomide alters key molecules in vascular development, including vascular endothelial growth factor VEGF, a key signaling molecule in vessel development (Yabu et al., 2005). Another study in zebrafish indicates thalidomide may affect gene expression patterns of *Shh* and *Fgf8* before vessels form in developing fins (Ito et al., 2010). However vascular markers, including VEGF, were not looked at in this study, and vessels in other regions of the embryo were not detailed. As zebrafish fins and chicken limbs develop and form differently, this could reaffirm that the drug behaves differently in different species.

Other studies have also demonstrated that blood vessels are targeted and destroyed by thalidomide (D'Amato et al., 1994; Hallene et al., 2006; Tamilarasan et al., 2006; Siamwala et al., 2012; Vargesson, 2013). These include rodent and rabbit corneal assays where blood vessels induced in response to a FGF soaked bead are destroyed following thalidomide application (D'Amato et al., 1994; Kenyon et al., 1997). Rat fetuses exposed to thalidomide in late gestation show blood vessel disruption in areas of the brain areas linked to causing autism (Hallene et al., 2006). Nitric oxide, a molecule required for endothelial cell function and blood vessel formation, appears to be protective of blood vessels in chicken embryos. Thalidomide inhibits nitric oxide expression resulting in vessel loss. Overexpression of nitric oxide can rescue and/or prevent thalidomide‐induced damage (Tamilarasan et al., 2006; Majumder et al., 2009; Siamwala et al., 2012). In zebrafish embryos, thalidomide causes blood vessel defects through VEGF receptor depletion (Yabu et al., 2005). Thalidomide has also been shown to successfully treat hereditary hemorrhagic telangiectasia (HHT) in human adult patients. The ‘leaky’ vessels seen in this condition are stabilized through recruitment of smooth muscle and inhibition of angiogenesis (Lebrin et al., 2010).

Other antiangiogenic drugs are teratogenic, for example, sodium valproate (Whitsel et al. 2002). In addition, loss of blood vessels in early development is usually lethal or can cause serious deformity/damage (Vargesson and Laufer, 2001; Vargesson, 2003, 2009).

Finally, many important molecules involved in embryonic vascular development and patterning have been reported to exhibit changed expression patterns following thalidomide exposure, for example, actin and tubulin, integrins, vascular endothelial growth factor, PDGFβ, nitric oxide, ceramide, angiopoietins, Notch, HIF, Slit2/Robo signalling and ROS (Stephens et al., 2000; Vargesson, 2009, 2013; Feng et al., 2014; Li et al., 2014.)

Taken altogether, the antiangiogenesis mechanism remains attractive and has a body of evidence supporting it.

## Cell death and ROS induction hypothesis

Another attractive hypothesis suggests Thalidomide can induce formation of ROS, which causes oxidative stress to cells, which has been proposed to cause limb defects (Parman et al., 1999; Hansen and Harris, 2004). By using an agent that traps and prevents oxidative stress intermediates, PBN – α‐phenyl‐N‐tertbutylnitrone), it has been demonstrated that following application to rabbits, it prevented thalidomide induced limb abnormalities in embryos (Parman et al., 1999) and prevented the formation of thalidomide hydrolysis products that caused teratogenesis in rabbit embryo cultures (Lee et al., 2011). Moreover, expression of *Fgf8* and *Fgf10* is lost in thalidomide treated rabbit embryos, but this effect is reversed when using the PBN spin trap reagent (Hansen et al., 2002). Thalidomide exposure can also induce cell death in limb tissue (Hansen and Harris, 2004; Knobloch et al., 2008, 2007; Parman et al., 2009; Wells et al., 2009) and could explain the loss of skeletal elements in the limb, as well as other damage to tissues in the body. Thalidomide upregulates bone morphogenetic protein (*Bmp*) expression and *Dickkopf‐1* (*Dkk1*) expression in the limb, which have important roles in limb development, among them regulating cell death (Knobloch et al., 2007). Use of PBN prevents this thalidomide‐induced upregulation and appears to protect the embryo from thalidomide‐induced damage (Knobloch et al., 2011, 2007). *Dkk1* is a downstream target of *Bmp*s and is an antagonist of *Wnt*, which also regulates cell survival and proliferation. Thus, induction of oxidative stress in embryonic tissue can cause thalidomide embryopathy through cell death induction (Parman et al., 1999; Hansen and Harris, 2004; Knobloch et al., 2008, 2007; Hansen and Harris, 2013). ROS induction also is linked to causing teratogenesis by other processes, eg maternal diabetes (Ornoy et al., 2015) and drugs, for example, ethanol, phenytoin (Knobloch et al., 2011; Hansen and Harris, 2013). Furthermore, ROS is required for normal embryogenesis in Xenopus and required for Xenopus tadpole tail regeneration, highlighting the important role ROS play in normal and causation of abnormal development (Love et al., 2011; Love et al., 2013). How ROS is involved in these processes remains unclear. Moreover, how thalidomide induces the cell death and ROS in specific tissues in a time sensitive manner and causes the range of damage observed is unclear. Cell death induction has also been observed in the forming limb following application of CPS49, an antiangiogenic analog of thalidomide, and perhaps inhibition of blood vessels could be a trigger or involved in induction of ROS and cell death (Therapontos et al., 2009; Vargesson, 2009).

The possible loss of blood vessels that could cause stress on cells resulting in cell death locally, and the evidence of the formation of thalidomide‐induced ROS *in vivo* can prevent/reduce thalidomide induced damage (Parman et al., 1999; Hansen et al., 2002; Hansen and Harris, 2013; Knobloch et al., 2007; Lee et al., 2011), suggest that both mechanisms together could explain much of the damage and range of damage seen in thalidomide embryopathy.

Other proposed mechanisms of thalidomide‐induced teratogenesis include effects on chrondrogenesis (Stephens et al., 2000), nerve toxicity/neural crest loss (McCredie and McBride, 1973), and DNA intercalation (Jonsson, 1972; Hague et al., 2011). All these proposed mechanisms likely have some involvement; however, in what order? We know that cartilage loss occurs in thalidomide treated animals and nerves are missing or changed, but these effects are likely downstream of the primary action of the drug, as both these tissues form relatively late in development (certainly after establishment and outgrowth of the major tissues including the limbs; Vargesson, 2003, 2013). One can envisage if tissue/organs are damaged or missing, that chrondrogenesis and nerve innervation would as a consequence be altered. Moreover, changes in vessel patterns could have an impact on chrondrogenesis, which requires a specialized vasculature to allow bone growth and remodeling (Kusumbe et al., 2014; Ramasamy et al., 2014). Indeed, there is evidence, in limbs of human embryos, of a vascular transition from an embryonic to an adult‐like state around day 45 postconception (∼59 days after last menstrual cycle; Hootnick et al., 1980; Levinsohn et al., 1991; Packard et al., 1991). Moreover, a failure of the correct transition of the vessel patterns has been proposed to explain a range of human lower limb abnormalities, including conditions such as short femur, which is associated with thalidomide embryopathy (Hamanishi, 1980; Hootnick et al., 1980; Levinsohn et al., 1991; Packard et al., 1991). Thus, damage to vessels in the forming limb could prevent or restrict the transition of the vascular pattern from the embryonic to the adult state, which could then impact bone formation and pattern.

Of course the big questions are what is/are the molecular target/s of thalidomide, and how does the drug cause such wide and ranging embryonic damage? Are there multiple mechanisms of action and/or targets?

## Molecular Targets of Thalidomide

Much is known about thalidomide's mechanism of action underlying its anti‐inflammatory and anti‐myeloma activities in human adults, than its teratogenic activities. Thalidomide inhibits TNF‐α expression rapidly, vital for the inflammatory response. However, new targets have been identified and linked to thalidomide teratogenesis, though how these targets cause the embryonic damage still remains unclear.

## Cereblon

Cereblon is a candidate gene linked to human mental retardation (Higgins et al., 2004; Ito et al., 2011). Cereblon is involved in ubiquitination and forms part of a Cullin4 (Cul4) containing E3 ubiquitination complex with DDB1 (DNA Binding Protein 1) which selects molecules for destruction (Ito et al., 2010; Ito et al., 2011). Thalidomide is thought to initiate teratogenesis by binding Cereblon, which presumably prevents Cereblon from establishing the E3 ubiquitination complex to target molecules for removal, causing mis‐regulation of developmental signaling molecules (Ito et al., 2010, 2011).

The role of Cereblon in thalidomide's anti‐inflammatory and antimyeloma response in adults is much better understood. For example, both Lenalidomide and Pomalidomide, structural variants of thalidomide used to treat a range of conditions, also interact with Cereblon and can selectively activate downstream targets Ikaros and Aiolos to be recruited for degradation and help mediate their action in multiple myeloma, further indicating that the anti‐tumor and teratogenic actions of the drug can be dissociated (Kronke et al., 2014; Lu et al., 2014). Elegant crystal binding structure assays have shown how thalidomide and Cereblon bind each other, that the S‐enantiomer of thalidomide (and also for both its related analogs, pomalidomide, and lenalidomide) can bind Cereblon, and also demonstrated how thalidomide can target myeloma cells and target them through binding Cereblon (Chamberlain et al., 2014; Fischer et al., 2014). Indeed these studies further suggest that several targets could be prevented from binding the E3 ubiquitination complex and thus not be degraded, including *Meis2*, a gene involved in proximal limb development (Fischer et al., 2014). Cereblon has also been shown to be able to bind Uridine, an essential component of the structure of DNA, and Uridine overexposure can reduce thalidomide‐like damage in zebrafish embryos (Hartmann et al., 2014). A study looking at *Cul4a*, which Cereblon interacts with to form the E3 ubiquitination complex, suggests in zebrafish that *Cul4a* may be able to bind to *Tbx5a*, a gene involved in pectoral fin (and forelimb) and heart development. This study adds to other studies suggesting that Cereblon following thalidomide binding could have tissue specific actions by binding tissue specific targets (Zhao et al., 2015). The ability of the Cereblon complex to induce the degradation of some proteins and prevent the degradation of others can help, at least in part, explain thalidomide's varied actions.

However, how Cereblon binding thalidomide actually results in teratogenesis, causes the range of embryonic damage and acts in the time‐sensitive window of action remains unclear. Following misexpression of a mutant Cereblon protein that can not bind thalidomide in embryos, thalidomide induced damage was reduced, which included *Fgf8* expression being maintained (Ito et al., 2010). Several studies have demonstrated that *Fgf8* signaling is reduced or lost in developing chicken and rabbit limbs, and zebrafish fins following thalidomide exposure (Hansen et al., 2002; Ito et al., 2010; Knobloch et al., 2011). However, it is unknown if regulation of *Fgf8* is directly or indirectly dependent on Cereblon expression. In addition the expression pattern of Cereblon in embryonic development is not fully understood. However, Cereblon knock out mice appear to be normal and fully functional (Lee et al., 2013). Altogether these studies demonstrate that Cereblon has important roles, which include metabolism regulation, brain development, intellectual ability, inflammatory response, and teratogenesis. Yet, Cereblon's precise role in embryonic development is still unclear and how it can cause teratogenesis by binding thalidomide is unclear presently. Moreover, how (or if) thalidomide binding to Cereblon fits with the drug's known antiangiogenic and cell death induction actions is also unclear. It is noteworthy that Cereblon has been reported to be expressed in the developing vasculature of the embryonic zebrafish head, potentially indicating a link between Cereblon and the antiangiogenic mechanisms of thalidomide action (Ito et al., 2010).

## Tubulin

An analog of thalidomide, 5HPP‐33, can bind tubulin, which was demonstrated through crystal structure binding assays (Rashid et al., 2015). Tubulin is part of the cytoskeleton, and,is required in cell proliferation, which is essential for angiogenesis and formation of new vessels in the embryo. This study demonstrated that tubulin is bound by the 5HPP‐33 thalidomide analog and cytoskeletal dynamics are altered preventing cell division. In the time‐sensitive window many organs/tissues are undergoing growth and maturation and could be affected in this manner, either directly or through loss of vessels, resulting in hypoxia and cell death. This work supports that of other studies which showed disruption of actin cytoskeleton dynamics in HUVEC cells following exposure to thalidomide (Tamilarasan et al., 2006) and an antiangiogenic analog of thalidomide, CPS49 (Therapontos et al., 2009), resulting in angiogenesis failure. Thalidomide binding to tubulin could prevent cell proliferation and migration, resulting in failed tissue morphogenesis, the severity of which could be time dependent depending on vascular and tissue maturation.

## Nitric Oxide and Soluble Guanyl Cyclase

Nitric oxide is involved in angiogenesis through stimulating the production of cGMP via activation of soluble Guanyl cyclase. cGMP levels are reduced following thalidomide administration, which causes failed angiogenesis in HUVEC cell cultures (Tamilarasan et al., 2006). When cGMP levels were increased this prevented thalidomide induced antiangiogenesis. *In silico* modelling indicates that thalidomide can bind soluble Guanyl cyclase (Majumder et al., 2009). This suggests that thalidomide can affect embryonic angiogenesis via inhibiting the nitric oxide signaling pathway.

In addition, several studies investigating molecular changes following thalidomide exposure in monkey fetuses and in human and mouse embryonic stem cells suggest changes in as many as 2000 gene expression profiles (Ema et al., 2010; Meganathan et al., 2012; Gao et al., 2014). Many of these gene expression profile changes are in vascular‐related and cytoskeletal‐related genes.

## Summary

Many gene expression changes result from thalidomide exposure, and seem linked to the vasculature and the cytoskeleton. Some binding partners of thalidomide have been identified, particularly Cereblon, Ikaros, and Aiolos, and play important roles in mediating thalidomide functions. Tubulin has been shown to bind a byproduct of thalidomide, which could explain the drug's anti‐angiogenic, anti‐proliferative and anti‐migratory actions. Moreover, several molecules have been found to protect against thalidomide‐induced damage in the embryo, including PBN, Nitric Oxide, Prostaglandin H Synthase and aspirin (Parman et al., 1999; Majumder et al., 2009; Lee et al., 2011; Arlen and Wells, 1996). These could act by preventing the parent molecule and/or the teratogenic thalidomide byproducts being produced or from acting. How these molecular targets and interactions of thalidomide actually cause and result in the range of damage seen in thalidomide embryopathy is still a matter of debate. Whether there are other targets of thalidomide, perhaps even tissue specific targets, also causing teratogenesis, remains an open question.

## Conclusions and Challenges

**Figure 5:**
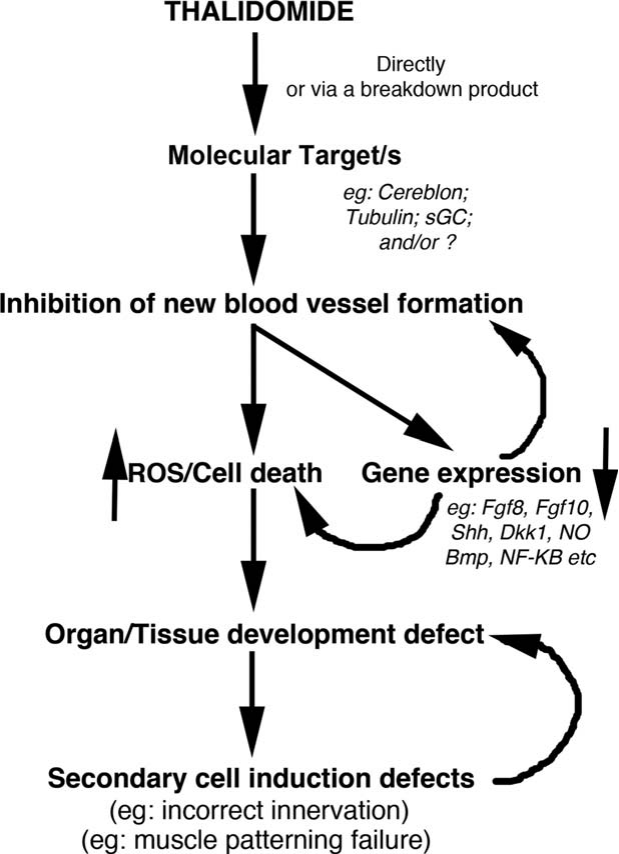
Framework of thalidomide induced embryonic damage. This framework incorporates the majority of the previously proposed models/hypotheses to attempt to provide an explanation for thalidomide embryopathy. Thalidomide and/or a breakdown product after binding a molecular target acts negatively on smooth muscle negative blood vessels, likely affecting the actin cytoskeleton of the endothelial cells, and preventing their proliferation and migration into avascular regions, causing oxidative stress, cell death, and gene expression loss, resulting in tissue damage. In rapidly developing tissues and organs, such as the limbs and internal organs, this would be devastating, causing tissue loss or tissue function loss, preventing growth. The damaged or missing tissues would then also fail to properly recruit and pattern proper chrondrogenesis, nerve innervation, muscle patterning, etc., exacerbating the condition and damage.

Thalidomide caused a disaster that still shocks the world today and sadly is happening again in Brazil. Exposure to the drug during early embryonic development resulted in severe and a range of damage never seen together before nor since. Indeed, arguably the most striking damage the drug caused is phocomelia, which still is not fully understood (Fig. 4). Other damage, such as radial dysplasia, internal organ damage, and genital injuries can occur in other syndromes, though, again in the case of radial dysplasia, how this comes about in general, let alone in thalidomide embryopathy, is unclear (Fig. 4). The fact that the drug has since been found to have many clinical uses, particularly for the treatment of leprosy and Multiple Myeloma (amongst others), ensures the drug is still used today. Yet, can a form of the drug ever be made that retains clinical effectiveness but not the side effect of birth defects? To be able to design new drugs for such a purpose, it is essential to understand how the drug caused the damage and in such a short time window. The mechanisms underlying thalidomide's teratogenic actions remain controversial. It is attractive to suggest that the drug targets multiple tissues through affecting a global process such as angiogenesis, possibly through affecting the actin cytoskeleton of rapidly proliferating endothelial cells and preventing their migration (Fig. 5). This would induce localized reactive species generation and cell death in specific tissues, and cause tissue damage. What are the targets in the endothelial cells? Tubulin? Could thalidomide bind specific types of endothelial cells? Recent work has identified a new H‐endothelial cell type found only in forming bones (Kusumbe et al., 2014; Ramasamy et al., 2014). Could thalidomide target tissue specific vessels, causing their loss through oxidative stress induction, cell death, failed growth, and loss of signaling? Alternatively, thalidomide could act in multiple tissues through different, tissue specific mechanisms, which could include antiangiogenesis, induction of reactive species, and of course through binding Cereblon and/or other molecular targets, including tubulin (Fig. 5). Ultimately, both possibilities are attractive and both could equally explain the severity and the wide range of damage that thalidomide causes in the embryo.

Despite a resurgence in interest in thalidomide as a clinical drug, its mechanisms of action to cause teratogenesis are still an enigma. However, given the great interest in thalidomide presently, it will hopefully only be a matter of time that we finally elucidate the full devastating mechanisms this drug induces.
